# Parents’ Education Anxiety and Children’s Academic Burnout: The Role of Parental Burnout and Family Function

**DOI:** 10.3389/fpsyg.2021.764824

**Published:** 2022-02-04

**Authors:** Kai Wu, Feng Wang, Wei Wang, Yongxin Li

**Affiliations:** Institute of Psychology and Behavior, Henan University, Kaifeng, China

**Keywords:** education anxiety, parental burnout, academic burnout, family function, moderated mediation model

## Abstract

This study aimed to explore the effect of parents’ education anxiety on children’s academic burnout, and the mediation effect of parental burnout and the moderating effect of family function. A total of 259 paired parents and children from two middle schools in central China participated in the survey. The questionnaire was conducted using the Educational Anxiety Scale, Parental Burnout Scale, Adolescent Student Burnout Inventory, and Family APGAR Index. Our results indicated that parental education anxiety had a positive predictive effect on children’s academic burnout. Moreover, parental burnout played a complete mediating role between parents’ education anxiety and children’s academic burnout. Finally, the relationship between education anxiety and parental burnout was moderated by family function, and higher family function buffered the effect of education anxiety on parental burnout. The results suggest the mechanism of parental education anxiety on children’s academic burnout, and the role of family function in alleviating parental burnout.

## Introduction

The traditional Chinese idiom of “Meng Mu San Qian”—which describes how a wise mother endeavors to find the best environment for her children’s education—shows that Chinese people have emphasized children’s education since ancient times. The imperial examination system has been implemented since the Sui and Tang Dynasties. While it provided opportunities for people from common backgrounds to attain official positions, it also strengthened the results-orientation of children’s educational evaluations. With dramatic shifts in parenting and environments, parents set progressively higher expectations for both their parenting behaviors and the development of their children, and are increasingly anxious about performing well as parents (Nelson and Nelson, 2010; Eibach and Mock, 2011; Lan, 2018). Thus, most parents typically expect their children to have a bright future.

The rapid development of the social economy has provided more parenting resources to raise children (Thomson et al., 1994; Hillman and Jenkner, 2004). However, high-speed development has also aggravated social competition and further raised parents’ expectations (Roskam et al., 2017). Specifically, parents increasingly worry about their children’s future and their failure in terms of education (Cheng, 2019). High parenting expectations and uncertainty regarding parenting outcomes have become common problems for Chinese parents. Recently, the popular TV series, “A Love for Dilemma,” incisively highlighted the phenomena of “educational involution,” and “pushing children to be the best.” The drama aroused extensive public discussion and illustrated the prevalence of education anxiety in contemporary China.

### Education Anxiety

Accordingly, the term “education anxiety,” which first emerged in news reports, has become familiar to the Chinese (Chen and Xiao, 2014), and has also been recognized by researchers (Cheng, 2019). For instance, Cheng (2019) indicated that education anxiety is a state of anxiety produced in the educational context. Regarding their children’s education, parent’s high expectations, uncertainty of education outcomes, and fear of failure may cause parents to experience tension, worry, panic, and other negative emotions. Furthermore, the content of parents’ education anxiety may encompass all aspects of their children’s development, such as whether their body height and shape meet certain standards, whether their learning motivation is strong, whether they have good learning habits, and whether they are skilled at interpersonal communication. That is, parents’ education anxiety can emerge during various developmental stages of their children and depend on children attaining the level of parents’ expectation of achievements.

Due to education being highly competitive in China, the quality of a child’s junior school education affects their ability to enter a prestigious high school, which also determines whether they can enter a well-known university. Therefore, parents may develop education anxiety when their children enter junior high school (Chang et al., 2020). These students are in their adolescence, and typically experience rapid physical and mental development, an awakening of self-consciousness, advanced independence, and critical thinking. However, they may also experience emotional instability and increased difficulty in communicating with their parents (Bailen et al., 2019). In addition, some students live in school dormitories, which further reduces opportunities for students to communicate with their parents (Zhang and Cao, 2018). Ultimately, a lack of communication between parents and children may worsen parents’ education anxiety.

### Relations Between Education Anxiety and Academic Burnout

Parents with education anxiety may have difficulties managing their expectations regarding their children. They may worry about providing sufficient educational resources for their children’s development, and the children achieving enough to meet their expectations. While students’ core task is learning (Chang et al., 2014), parents’ anxiety mainly revolves around their children’s studies. For example, parents may worry about their children’s learning motivation, habits, and performance. Furthermore, parents’ negative emotions were related to a decrease in positive parenting behavior and an increase in negative parenting behavior (Dallaire et al., 2006). Accordingly, these anxieties are unproductive in children’s education and may further increase pressure on children regarding their learning, resulting in academic burnout (Cheng, 2019).

Academic burnout can be regarded as an extension of career burnout as students’ routines may include structured activities, such as attending class and submitting assignments, which can be considered “work” (Lin and Huang, 2014). Researchers have indicated that emotional exhaustion, depersonalization, and feelings of low personal accomplishment can be experienced in students’ learning process, course stress, course load, and so forth (Balogun et al., 1996; Lingard et al., 2007). Walburg (2014) reviewed the academic burnout in high school students and confirmed the three-dimensional construct of academic burnout. Furthermore, academic burnout was positively related to depression and school dropout. School pressure, peer groups, and school engagement were risk factors for academic burnout.

According to social learning theory (Bandura, 1989), children learn their parents’ reaction patterns. Anxious parents may show tension, or disgust when encountering pressure and frustrating situations. When children experience stressful situations, they may also exhibit a series of negative symptoms that imitate their parents’ reaction patterns that produce tension, anxiety and disgust. Specifically, students may lose their enthusiasm for studying and school activities, may have difficulty communicating with their classmates or friends, and may fail in obtaining a sense of achievement from their studies (Wu et al., 2007). Previous studies have also indicated the positive relationship between parents’ education anxiety and their children’s academic burnout (Cheng, 2019). Therefore, in line with prior studies, Hypothesis 1 was proposed: parental anxiety is positively related to academic burnout.

### Relationship Between Education Anxiety and Parental Burnout

Parental burnout refers to a group of negative symptoms caused by long-term parenting stress, such as emotional exhaustion related to the parental role, self-comparison with previous self, being fed up of their parental role, and emotional distance from children (Roskam et al., 2018). According to the balance between risks and resources (BR^2^) theory (Mikolajczak and Roskam, 2018), parental burnout stems from high parenting requirements (perfectionism in parenting, low emotional intelligence, poor parenting habits, and lack of support from family and partners, etc.) and limited parenting resources (parents’ self-empathy and lack of emotional support, etc.). Therefore, the emergence of parental burnout results from inadequate parenting resources, which cannot meet demand requirements. Moreover, high expectations regarding education (high parenting requirements) and anxieties that there are insufficient educational resources to meet children’s needs (limited parenting resources) are predominant sources of education anxiety (Cheng, 2019). Thus, if parental burnout results from the long-term imbalance between resources and demands, then parenting anxiety may be an antecedent of their burnout. Therefore, Hypothesis 2 was proposed: parental anxiety is positively related to parental burnout.

### The Relationship Between Education Anxiety and Academic Burnout

Furthermore, both the concepts of parental burnout and academic burnout are derived from job burnout (Pines et al., 1981; Roskam et al., 2017). Although parental burnout focuses on parents’ inner experiences in the parenting process (Roskam et al., 2018), and academic burnout focuses on children’s subjective feelings in the learning process (Farina et al., 2020), both concepts overlap considerably. Based on the social learning theory (Bandura, 1989), parents are usually their children’s first teachers, role models, and main learning objects. Children acquire knowledge from their parents and learn their behaviors and emotional expressions (Bian et al., 2016). When children notice their parents’ negative emotional experiences (parental burnout), they may learn from their parents’ expressions and reproduce similar emotions (academic burnout) in the corresponding academic environment. Therefore, parental burnout may be an antecedent to children’s academic burnout. Combined with a previous study (Cheng, 2019) and Hypothesis 2, Hypothesis 3 was proposed: the relationship between parents’ education anxiety and children’s academic burnout is mediated by parental burnout.

### The Moderation Effect of Family Function

According to the BR^2^ model (Mikolajczak and Roskam, 2018), excessive parental requirements are not necessarily related to parental burnout. Specifically, when parenting resources are insufficient to meet parenting requirements, anxious parents may not have extra resources to cope with their anxiety. In this situation, education anxiety may positively related with the occurrence of parental burnout; however, when parents have sufficient resources to cope with their requirements, they may avoid the occurrence of parental burnout. Therefore, the relationship between parenting requirements and parental burnout may be moderated by parenting resources. Family function reflects the intimacy, mutual support, and cooperation among family members, and could also be used to measure individual social support and emotional support (Zhang, 2013). In addition, family function was considered closely related to an individual’s psychological status (Beavers and Voeller, 1983) and a well-functioning family can improve the care of family members and promote the self-esteem and self-acceptance ability of children (Bao, 2019). Therefore, high family function means that families can provide important emotional and social support (Danzeng et al., 2015), and it constitutes a typical parenting resource.

Although raising children is the responsibility and obligation of both spouses (Cheng et al., 2021), spouses may have different parental roles. Specifically, the role of primary caregivers may be more important than that of secondary caregivers. When the primary caregiver faces high parenting requirements and experiences education anxiety, high family function from the secondary caregiver or social support from other family members may reduce their parenting pressure and enrich parenting resources. Prior studies have already shown that social support can decrease the level of parental burnout (Ardic, 2020; Szczygiel et al., 2020). Moreover, partner support not only reduces parenting pressure, but also helps to maintain the balance between parenting demands and resources (Séjourné et al., 2012; Parfitt and Ayers, 2014). Therefore, Hypothesis 4 was proposed: the relationship between parenting anxiety and parental burnout is moderated by family function.

### Present Study

**FIGURE 1:**
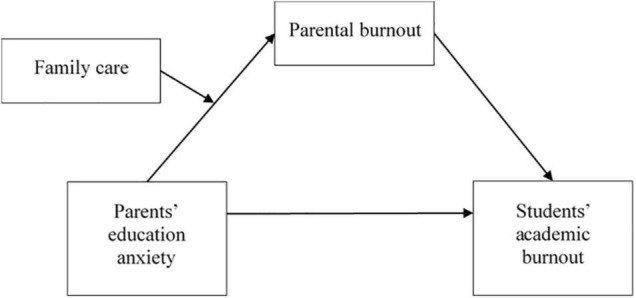
The framework of the present study.

Thus, based on the BR^2^ theory, the current study aimed to examine the effects and mechanisms of parents’ education anxiety on children’s academic burnout. Paired data from primary caregivers and children were utilized. Parental burnout was set as a mediating variable and family function as a moderating variable, and a moderated mediation model was built. The research framework is illustrated in Figure 1.

## Materials and Methods

### Sample

Data were collected through convenience sampling. Participants were junior middle school students and their primary caregivers (father or mother) from two middle schools in Central China. Two versions of the questionnaire (students’ and parents’ versions) were distributed to students during class. Parents were asked to answer the education anxiety, parental burnout, and family function questionnaire. Children were asked to answer the academic burnout scale. After students completed their version in class, they delivered the parents’ version (which was sealed in an envelope) to their primary caregivers at home. Upon completion, the primary caregivers sealed the questionnaire in the reply envelope and returned it to the students, who subsequently returned the questionnaires to school. Participants were required to provide signed informed consent which detailed that participation in the survey was voluntary, refusal to participate would not incur any repercussions, and that participants could withdraw at any time. All students provided informed consent to participate in the study, and their participation in the study was also approved by their parents. This survey was approved by the research ethics committee of the authors’ academic institution.

Both the children’s and parents’ questionnaires contained a question, “Who is your primary caregiver?” and “Who is the primary caregiver of the child?” respectively. The students chose a response from father, mother, or others, while parents selected either themselves, their spouse, or others. After both questionnaires were returned, we checked whether the data matched. If the answers of parents and students about the primary caregiver were different, the paired questionnaires were excluded. If the answer of the primary caregiver was “others,” these questionnaires were also excluded. In total, 259 pairs of data were retained for subsequent statistical analysis. Regarding primary caregivers, there were 206 mothers (79.54%) and 51 fathers (20.46%); the average age was 40.47 (*SD* = 3.76) years. For educational level, 123 had below high school education, 126 had higher than undergraduate education, and 10 did not answer. Regarding students, there were 118 boys (45.56%), 132 girls (50.97%), 9 missing (3.47%), 129 from grade 7 and 130 from grade 8; the average age was 12.89 (*SD* = 0.69) years.

### Measures

#### Parents’ Education Anxiety

Parents’ education anxiety was measured using the Parental Anxiety about Children’s Education Questionnaire (Cheng, 2019). It includes three dimensions: achievement anxiety, ability anxiety, and health anxiety; each dimension has four items, totaling 12 items. An example item is: “I am worried that my child will not have a stable job in the future.” All items were assessed on a 5-point Likert scale ranging from 1 (“never”) to 5 (“always”). Cronbach’s α was 0.872 in the current study.

#### Parental Burnout

Parental burnout was measured using the Chinese version of the Parental Burnout Assessment (PBA; Cheng et al., 2020). This was translated from the English version of Roskam et al. (2018) and has satisfactory reliability and validity. It comprises 21 items; an example item is: “I feel as though I’ve lost my direction as a dad/mum.” Each item was rated using a 7-point Likert scale, ranging from 1 (“completely inconsistent”) to 7 (“completely consistent”), with a higher score representing higher burnout. Cronbach’s α was 0.927 in the current study.

#### Family Function

Family function was measured using the Chinese version of the Family Adaption Partner Growth Affection Resolve Index (family APGAR index; Chen et al., 1980). It was translated from the English version of Smilkstein (1983) and has satisfactory reliability and validity. It includes five dimensions of adaption, partner, growth, affection and resolve, and each dimension has one item. An example item includes: “Family resources are available for coping.” Each item was rated on a 3-point Likert scale, ranging from 0 (“rarely”) to 2 (“always”). Cronbach’s α was 0.862 in the current study.

#### Academic Burnout

Academic burnout was measured using the Adolescent Academic Burnout Scale (Wu et al., 2010). The scale includes three dimensions: low sense of achievement, academic alienation, and physical and mental exhaustion. It consists of 16 items, for example: “I cannot feel any sense of achievement from learning” for low sense of achievement; “I am so bad at my study that I really want to give up” for academic alienation; and “I feel extremely tired at the end of a day studying” for physical and mental exhaustion. Each item was rated on a 5-point Likert scale, ranging from 1 (strongly inconsistent) to 5 (strongly consistent). Some items were reverse scored. A higher score on the scale indicates a higher level of academic burnout. The Cronbach’s α was 0.877 in the current study.

#### Demographic Variables

Demographic items, including parents’ age, gender, education level, and their child’s gender and age were recorded.

### Data Analysis

All data analyses were conducted using IBM SPSS 23.0, with a process macro. First, common method variance was examined using confirmatory factor analysis. Correlation analyses were then conducted to preliminarily examine the hypotheses. Finally, the mediation effects of parents’ education anxiety and the moderating effects of family function were examined via regression and bootstrap analyses.

## Results

### Common Method Bias

Although the present study adapted paired data, the answers were derived from different resources. All questionnaires were self-assessment scales that may produce common method bias. Therefore, common method bias was examined using the Harman single factor test (Zhou and Long, 2004). The results of the exploratory factor analysis showed that the number of factors without rotation was greater than 1, and the variance interpretation percentage of the first principal component was 23.62%, less than 40%. This indicated that common method bias had little effect on the overall results of the present study (Ashford and Tsui, 1991). Furthermore, common method variance was examined by controlling for the effects of the unmeasured latent method factor (Podsakoff et al., 2003). In the confirmatory factor analysis model, each item was allowed to load on its respective construct (i.e., education anxiety, parental burnout, family function, and academic burnout). In addition, the common method variance factor was created, with all items allowed to load. The latent factor did not correlate with other factors. The variance explained by the latent method factor was 2.4%, which is lower than the median of 25% reported in a previous study (Williams et al., 1989). These results provide further evidence that common method variance had little effect on the overall results of the present study.

### Descriptive Statistics and Correlation Analysis

Firstly, a series of the independent-samples *t* test were conducted to examine whether the demographic characters could affect the variables. The results showed that education anxiety, parental burnout (*t* = −0.96, *df* = 251, *n*.*s*.), and family function (*t* = 1.46, *df* = 251, *n*.*s*.) did not show significant differences on parents’ gender (for education anxiety: *t* = −0.08, *df* = 251, *n*.*s*.; for parental burnout: *t* = −0.96, *df* = 251, *n*.*s*.; for family function: *t* = 1.46, *df* = 251, *n*.*s*.) or education level (for education anxiety: *t* = −0.08, *df* = 251, *n*.*s*.; for parental burnout: *t* = −0.96, *df* = 251, *n*.*s*.; for family function: *t* = 1.46, *df* = 251, *n*.*s*.). In addition, academic burnout did not show significant differences on students’ academic burnout (*t* = −0.72, *df* = 248, *n*.*s*.) (Table 1).

**TABLE 1:** Descriptive statistics and correlations.

		*M*	*SD*	➀	➁	➂	➃	➄	➅	➆	➇	➈	➉
➀	Education anxiety	3.05	0.78	(0.872)									
➁	Achievement anxiety	3.10	1.01	0.88**	(0.887)								
➂	Ability anxiety	2.74	0.91	0.81**	0.52**	(0.676)							
➃	Health anxiety	3.37	0.90	0.75**	0.50**	0.51**	(0.698)						
➄	Parental burnout	1.67	0.75	0.45**	0.42**	0.32**	0.36**	(0.927)					
➅	Academic burnout	2.32	0.61	0.21**	0.19**	0.24**	0.06	0.23**	(0.877)				
➆	Physical and mental exhaustion	2.66	0.89	0.100	0.06	0.17**	-0.01	0.18**	0.76**	(0.752)			
➇	Academic alienation	1.66	0.72	0.21**	0.19**	0.24**	0.06	0.28**	0.83**	0.57**	(0.826)		
➈	Low sense of achievement	2.60	0.71	0.20**	0.19**	0.19**	0.09	0.13*	0.83**	0.37**	0.51**	(0.829)	
➉	Family function	2.45	0.51	−0.20**	−0.20**	−0.17	−0.09	−0.27**	−0.25**	−0.16**	−0.22**	−0.23**	(0.862)

*p < 0.05 and **p < 0.01.

### Moderated Mediation Models

Aligned with Wen and Ye’s (2014) suggestion, a multiple regression analysis was conducted to examine the direct effect of parents’ education anxiety on children’s academic burnout, the mediation effect of parental burnout, and the moderation effect of family function. In the first model, academic burnout was set as the dependent variable and education anxiety as the independent variable. In the second model, parental burnout was added as an independent variable. In the third model, parental burnout was set as the dependent variable, and education anxiety, family function, and interaction of family function and education anxiety were set as independent variables. The results were shown in Table 2.

**TABLE 2:** Results of multiple regression analyses.

	Model 1		Model 2		Model 3	
	Academic burnout		Academic burnout		Parental burnout	
	β	*t*	β	*t*	β	*t*
Education anxiety	0.21	3.45**	0.13	1.96^+^	0.46	8.18***
Family function					–0.14	−2.56*
Education anxiety × Family function					–0.20	−3.68***
Parental burnout			0.17	2.57*		
*R* ^2^	0.04		0.07		0.27	
*△R^2^*	0.04		0.03		0.27	
*F*	11.91**		9.39***		31.958***	

*The dependent variables were academic burnout.*

*^+^p < 0.10.*

*p < 0.05; **p < 0.01; ***p < 0.001.

Parents’ education anxiety had a positive effect on children’s academic burnout (β = 0.21, *t* = 3.45, *p* < 0.01), which further supported Hypothesis 1. When parental burnout was added to the model, parental burnout had a significant effect on academic burnout (β = 0.17, *t* = 5.57, *p* < 0.05). Education anxiety was less significant than Model 1 (β = 0.13, *t* = 1.96, *p* < 0.10), which preliminarily supported Hypothesis 2. In addition, parents’ education anxiety (β = 0.46, *t* = 8.18, *p* < 0.001), family function (β = −0.14, *t* = −2.56, *p* < 0.05), and the interaction between education anxiety and family function (β = −0.20, *t* = −3.68, *p* < 0.001) were significantly related to parental burnout, which preliminarily supported Hypothesis 3.

To further examine the hypothesized moderated mediation model, Model 14 of the process macro (Hayes, 2013) was used. Parents’ education anxiety was set as the independent variable, parental burnout as a mediating variable, family function as a moderating variable, and students’ academic burnout as the dependent variable. Following the suggestions of Fang et al. (2014), a non-parametric bootstrapping method (n = 5,000) was used, with a 95% confidence interval calculated using the bias-corrected bootstrapping method. The results showed that the moderated mediation model was significant (the indexes of moderated mediation were -0.015, *SE* = 0.010, 95% CI [−0.042, −0.002]. Table 3 shows the mediating effects of parental burnout under different levels of family function.

**TABLE 3:** The mediation effects of parental burnout under different levels of family function.

	Family function	*EFFECT*	*SE*	*95%CI*
Parental burnout	*M-SD*	0.122	0.062	[0.016, 0.264]
	*M*	0.083	0.039	[0.012, 0.168]
	*M* + *SD*	0.044	0.021	[0.009, 0.094]

**FIGURE 2:**
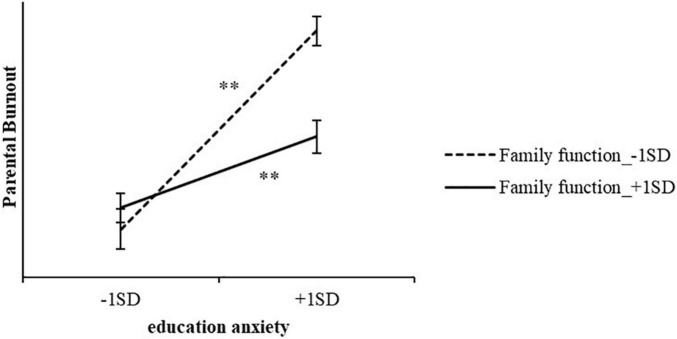
The moderation effects of family care.

A simple slope test was conducted to further explore the moderating effects of family function (Figure 2). Education anxiety showed significant positive effects on parental burnout, regardless of whether family function was high or low. However, with low family function, the predictive effect of education anxiety on parental burnout (simple slope = 0.15, *t* = 7.57, *p* < 0.001) was higher than that in high family function (simple slope = 0.12, *t* = 3.36, *p* < 0.001). These results suggest that family support could help primary caregivers cope with the negative effect of education anxiety, and provided further support for Hypothesis 3.

## Discussion

At present, parents focus on and invest more resources in children’s education with high expectations. In addition, because of uncertainties regarding educational outcomes, parents’ education anxiety is becoming more prevalent. Previous studies indicate that education anxiety not only negatively affects parents’ sleep quality, but also exacerbates children’s academic burnout (Cheng, 2019). However, few works have focused on this effect from the children’s perspective and examined the mechanism. Therefore, by using paired data, the present study established a moderated mediation model, and examined the mediation effect of parental burnout and the moderation effect of family function. The result generally supported the hypotheses.

Although the term “education anxiety” often appears in various news reports, there are few empirical studies on this social phenomenon (Li, 2020). The present study examined the relationship between education anxiety and parental burnout based on the characteristics and current context of family education in China. The results support that parents’ education anxiety could be positively related to their level of parental burnout. This result is consistent with the BR^2^ theory of parental burnout (Mikolajczak and Roskam, 2018). Furthermore, parents’ education anxiety may partly originate from perfectionism, and perfectionism could also be an antecedent of parental burnout (e.g., Meeussen and Van Laar, 2018; Furutani et al., 2020). Future studies should control the effects of perfectionism, and examine the relationship between education anxiety and parental burnout.

According to the BR^2^ model, anxiety about high educational expectations, concerns about the uncertainty of educational achievements, and fear of educational failure, could be regard as parenting requirements. When these requirements exceed parenting resources, it may lead to resource depletion, resulting in parental burnout. The present study provides a new perspective for exploring the antecedents of parental burnout. Therefore, the prevention and intervention of parental burnout may be achieved by decreasing education anxiety. Parents should parents should assume a scientific approach toward parenting, adjust their parenting expectations, and set realistic parenting goals to reduce education anxiety and parental burnout.

An essential aspect of parents’ education behavior is the interaction between parents and children (Guo, 2011). Parents with high levels of anxiety may find it difficult to adjust their behaviors. Combined with high expectations, these parents may emphasize the learning of knowledge and skills, and ignore their children’s emotional demands. This results in improper rearing behavior, for instance, parents may permit their children to participate in many tutorial classes and reduce their rest and entertainment time. Children may not realize their parents’ expectations; thus, negative parental behavior may harm the parent-child relationship and result in parent-child conflict (Han, 2018). In addition, children become vulnerable in parent-child relations, and may suffer serious psychological damage.

The present study examined whether the relationship between education anxiety and children’s academic burnout could be mediated by parental burnout. The results provide new evidence to explain the mechanism of education anxiety on children’s academic burnout. Furthermore, this aligns to prior studies that suggest job burnout and marriage burnout are positively correlated (Pines et al., 2011; Dacey, 2019). The present study contributed new evidence that different kinds of burnout may be correlated with each other. Future studies should adopt a more comprehensive perspective, and examine the relationship between parental burnout and couple burnout from a family perspective, job burnout from a work perspective, and academic burnout from children’s developmental perspective.

According to social learning theory (Bandura, 1989), parents serve as role models and are the main learning objects for their children. Children not only observe their parents’ behaviors, but also capture their parents’ emotional reactions. Children internalize the pattern of their parents’ behavior and emotional reactions within their psyche and reproduce them in appropriate situations (Bandura, 1989; Bian et al., 2016). Parents suffering from burnout tend to exhibit a series of negative symptoms while interacting with their children, such as psychological alienation, emotional apathy, and escape from responsibility (Roskam et al., 2018). Children observe and learn these reactions which may manifest in significant daily activities. Specifically, in the education context, children may display corresponding symptoms of academic burnout. Therefore, burnout symptoms are transmitted from parents to children.

From a BR^2^ model perspective, parental burnout is the result of a long-term imbalance between parenting requirements and resources. Therefore, parenting resources play a moderating role in the relationship between parenting requirements and parental burnout. Family is not only the initial and primary platform for parenting activities (Cheng et al., 2021), but also serves as the foundation of intimate relationships, partner communication, and parent-child interactions (Liu et al., 2018). Therefore, families may provide the main resources for parenting. Given that high family function represents ideal family functioning (Lv and Gu, 2005), it could be regarded as a typical parenting resource. The present study examined its moderating effect on the relationship between education and parental burnout. The results showed that, compared with low family function, individuals with high family function showed less parental burnout with increased education anxiety. These findings suggest that to avoid parental burnout, individuals should strive to enrich their own parenting resources, and should seek help from other family members when they experience anxiety.

In addition, the present study may also contribute in practical ways. From a parental perspective, parents should adhere to scientific parenting goals to avoid becoming anxious. Meanwhile, they should remain alert for signs of anxiety or burnout and avoid transmitting these emotions to their children. Family therapists should focus on establishing and improving the overall family function of anxious parents and ensure sufficient support from their family, partners, or other members.

### Limitations and Future Directions

This study had several limitations. First, present study adopted paired data collection and confirmation of the primary caregivers. The cross-sectional nature of the study limited our results to proving the causal relations between variables. Future studies should adopt a longitudinal research design to collect data at multiple time points, which may enable a more in-depth examination of the relationship between variables. Second, junior high school is a critical period in children’s education when their physical and psychological characteristics are undergoing rapid development. Adolescents and their parents are thus, ideal targets for studying education anxiety, parental burnout, and academic burnout. However, parenting activities constitute long-term interactions. Moreover, infants, children, and adolescents of different ages have varying physical and mental development tasks, and their parents also have distinct parenting pressures. Therefore, parents may have varying levels of education anxiety or parental burnout during children’s various developmental stages. The present study only included junior middle school students and their parents, and whether these results can be extended to children at other stages of development and their parents must be further explored. Third, the present study mainly focused on the general effect of education anxiety on academic burnout, we failed to examine the relationship between each sub-dimension of academic anxiety and academic burnout. Future studies should focus on these issues, and conduct meticulous designs to explore the relationship between each sub-dimension.

## Conclusion

In general, by establishing a moderated mediation model, the present study confirmed that parents’ education anxiety could be associated with children’s academic burnout. In addition, education anxiety may be an antecedent of parental burnout. The different forms of burnout could be associated with each other.

## Data Availability Statement

The datasets generated for this study are available on request to the corresponding author.

## Ethics Statement

The studies involving human participants were reviewed and approved by the Research Ethics Committee of the Institute of Psychology and Behavior, Henan University. Written informed consent to participate in this study was provided by the participants’ legal guardian/next of kin.

## Author Contributions

YL and KW generated the idea and designed the study. FW performed material preparation and data collection. FW and WW performed data analysis. WW wrote the first draft of the manuscript. All authors commented on previous versions of the manuscript, read, and approved the final manuscript.

## Conflict of Interest

The authors declare that the research was conducted in the absence of any commercial or financial relationships that could be construed as a potential conflict of interest.

## Publisher’s Note

All claims expressed in this article are solely those of the authors and do not necessarily represent those of their affiliated organizations, or those of the publisher, the editors and the reviewers. Any product that may be evaluated in this article, or claim that may be made by its manufacturer, is not guaranteed or endorsed by the publisher.
